# Health workers’ knowledge, perceptions, and practices relating to antimicrobial use and resistance in cross river state, Nigeria: a cross sectional study

**DOI:** 10.1186/s41043-026-01274-1

**Published:** 2026-04-11

**Authors:** Akaninyene Asuquo Otu, Obiageli Chiezey Onwusaka, Ubong Aniefiok Udoh, Ugbe Maurice Joel Ugbe, Emmanuel Edet Effa, Joel Inyang, Bassey Ebenso, Fatumo Abdi, Chikezie Okwesileze, Bassey Ewa Ekeng, Ita Okokon Ita, John Walley

**Affiliations:** 1 Nuffield Centre for International Health and Development, University of Leeds, Leeds, UK; 2 Faculty of Public Health, 4 St. Andrews Place, London, NW1 4LB UK; 3 Foundation for Healthcare Innovation and Development (FHIND), Calabar, Cross River State Nigeria; 4 Department of Public Health, Faculty of Allied Medical Sciences, College of Medical Sciences, University of Calabar, Calabar, Cross River State Nigeria; 5 Department of Medical Microbiology and Parasitology, Faculty of Medicine, College of Medical Sciences, University of Calabar, Calabar, Cross River State Nigeria; 6 Department of Internal Medicine, University of Calabar, Calabar, Cross River State Nigeria; 7 University of Calabar Teaching Hospital, Calabar, Cross River State Nigeria

**Keywords:** Health workers, Antimicrobial use, Antimicrobial resistance, Antimicrobial stewardship, Knowledge, Perceptions and practices, Cross sectional survey

## Abstract

**Introduction:**

Antimicrobial resistance (AMR) is a serious global health issue with health workers (HWs) being central to addressing this issue. This study aimed to assess the knowledge, perceptions, and practices of HWs regarding antimicrobial use/AMR in Cross River State, Nigeria.

**Methodology:**

This cross-sectional survey involved 431 HWs in randomly selected healthcare facilities (13 primary and 23 secondary facilities) in Cross River State. Data were collected using a structured questionnaire. Descriptive and inferential analysis were performed using SPSS version 23.

**Results:**

Of 431 HWs approached, 427 (99.1%) responded. Nurses comprised the largest group (37%), followed by medical doctors (26.5%). Overall, 60% of the respondents demonstrated good knowledge 30% demonstrated moderate knowledge, and 10% demonstrated poor knowledge of AMR and antimicrobial use. Perceptions were positive, with 81% categorized as good. Practices were good in 65% (of consenting HWs although 54.3% (*n* = 232) admitted to prescribing antibiotics not in accordance with guidelines. Identified gaps included prescribing due to patient pressure (47%) and stock availability (66%). Professional role (χ² = 41.72, df = 18, *p* = 0.001) and years of experience (χ² = 26.78, df = 10, *p* = 0.013) were significantly associated with good knowledge. Also, professional role (χ² = 28.41, df = 9, *p* = 0.001) and years of experience χ² = 14.67, df = 5, *p* = 0.012) were significantly associated with good practices. No statistically significant associations were found with perceptions.

**Conclusion:**

HWs in Cross River State exhibited predominantly good knowledge and perceptions of AMR, but practices, though good, revealed areas for improvement, particularly in guideline adherence and resistance to external pressures.

## Introduction

Antimicrobial resistance (AMR) represents one of the most pressing threats to global health security, rendering once-effective treatments ineffective against common infections and potentially reversing decades of medical progress [1, 2]. The World Health Organization (WHO) has recognised AMR as a global public health concern, as it makes infectious diseases harder to treat and results in increased mortality rates [3]. Recent estimates have revealed that bacterial AMR directly caused 1.27 million deaths in 2019, and indirectly caused 4.95 million deaths, with projections estimating 1.9 million attributable and 8.22 million associated deaths annually by the year 2050 unless addressed [3]. This crisis is exacerbated in low- and middle-income countries (LMICs), due to low infection prevention and control, over-the-counter availability of antibiotics, low diagnostic capacity driving the emergence of resistance and poor national surveillance systems coverage in African countries [4, 5]. Apart from health impacts, AMR is predicted to exacerbate economic costs, with the potential of driving millions of individuals into poverty due to prolonged illness and higher healthcare costs [6, 7].

In Nigeria, AMR is exacerbated by widespread antimicrobial misuse in animals, agriculture and humans, with sub-optimal antimicrobial prescription in healthcare facilities and low community awareness [8–10]. Although national surveillance data remain limited, available reports indicate high resistance among priority pathogens e.g., MRSA and ESBL-producing Enterobacterales in Nigeria. In 2019, Nigeria had an estimated 263,000 deaths associated with bacterial AMR [8]. The drivers of AMR are improper dosing regimens, self-treatment, and poor sanitation, which encourage the spread of resistance [8]. Nigeria’s National Action Plan for AMR (2024–2028) calls for multisectoral interventions, but gaps in implementation such as a lack of proper training for health workers continue to exist [11].

Health workers (HWs), such as doctors, nurses, pharmacists, and community health extension workers (CHEWs), play crucial roles in preventing AMR through appropriate prescribing, patient counseling, and compliance with antimicrobial stewardship (AMS) principles [12]. In LMICs like Nigeria, most antibiotic prescriptions are made in primary care units and HWs’ knowledge, attitudes, and practices drive resistance patterns [13]. Levels of awareness in Nigeria are low due to resource and educational deficits. A national survey showed that half (85.3%) of the HWs were aware of AMR, but only 49.2% of them had good knowledge of AMR [14]. Another study in Nigeria reported that out of the majority of physicians who were aware of AMR, only 22.3% were knowledgeable about antibiotic prescriptions [15]. A study in Delta State of Nigeria reported that 58% of 420 adult respondents had poor awareness of AMR [16]. 

Nigeria’s 2024–28 National AMR Action Plan explicitly calls for strengthening health-worker capacity (including better training on antimicrobial use) as a priority. Assessing health‐worker knowledge, attitude and practice with respect to antimicrobial use is therefore essential to tailor interventions. This kind of evidence will guide context‐specific policies and training programmes that will bolster AMS and help curb AMR. Such evidence is scarce from the South-South zone of Nigeria where Cross River State (CRS) is located and represents a gap in the existing literature.

This study evaluated the knowledge, perceptions, and practices relating to antimicrobial use and resistance among HWs in CRS to identify gaps and suggest evidence-based strategies for enhancing AMS delivery in resource-limited settings. The working hypothesis was that there was no statistically significant association between HWs’ socio-demographic characteristics and their knowledge, perceptions, and practices of antimicrobial use and AMR.

## Methodology

### Study setting

CRS has a population of 3,866,300 people who are served by 1,028 publicly owned health facilities made up of 3 tertiary level facilities, 16 secondary level facilities (general hospitals) and 909 primary level facilities comprising 722 health posts, 269 primary health centres (PHCs) and 18 comprehensive health centres (CHCs). Approximately 70% of clients in CRS access care from public facilities where antibiotics are prescribed by doctors, nurses and CHEWs [17]. CRS is divided into North, South and Central zones, each with six Local Government Areas (LGAs). TheseLGAs are further divided into political wards and then communities.

### Study design

A cross-sectional survey was conducted from November to December 2023 in CRS, Nigeria. HWs from primary, secondary and tertiary healthcare facilities were selected using stratified random sampling.

The sample size for the study was determined using the Bluman’s (2004) formula i.e.:

$$n=Z^{2}pq$$

$$d^{2}$$

where n = sample size for the quantitative study approach.

Z = level of confidence which was 1.96 (i.e. 95% confidence interval).

P = Prevalence of knowledge of AMR was 41% (Auta et al., 2014).

$$q=1-p = (1-0.41)$$

d = level of precision or (non-response rate) which was 5% (0.05).

Therefore, the sample size was:

$$n=1.96^{2}\times 0.41(1-0.41)$$

$$0.0025$$

$$=371$$

The sample size was increased by 10% in order to create room for non-response (37.17).

Therefore, sample size was approximately 408.

## Sampling procedure

In the North and South senatorial zones, two out of six LGAs were randomly selected, while in the more populous Central zone three out of six LGAs were selected.

**Fig. 1:**
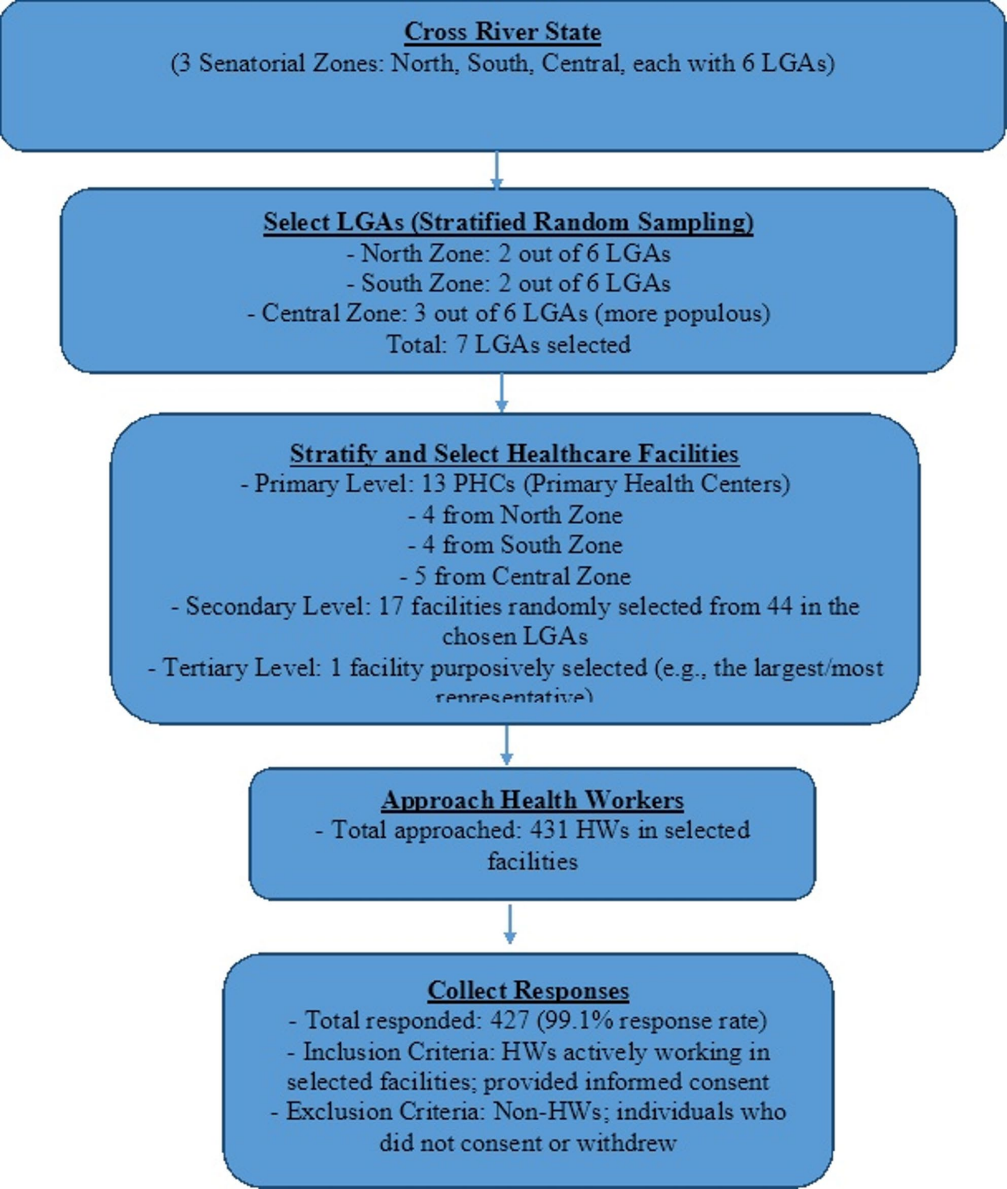
Diagrammatic presentation of the sampling procedure

Healthcare facilities were stratified into primary, secondary, and tertiary levels. A simple random sampling technique was used to select four PHCs from the Northern and Southern senatorial districts, and five PHCs from the Central senatorial district, making a total of thirteen PHCs. Seventeen secondary-level facilities were randomly picked from the forty-four facilities in the selected LGAs. One of the three tertiary healthcare facilities was purposively selected, as it is the largest and most representative of tertiary care in the state, ensuring inclusion of high-level HWs. To ensure objectivity using the simple random sampling method, each facility was assigned a unique identification number, and the required number of facilities was selected using a computer-generated random number sequence. This process ensured that every eligible facility had an equal probability of selection. In each selected healthcare facility, all eligible health workers present during the study period were approached face-to-face by trained data collectors and invited to participate in the study. Recruitment was not targeted to specific cadres; rather, all consenting eligible health workers were invited to participate. Figure 1 below provides a diagrammatic view of the procedure.

### Instrument for data collection

The structured questionnaire was developed by reviewing relevant literature on knowledge, attitudes, and practices (KAP) regarding antimicrobial use and resistance among healthcare workers in low- and middle-income countries. It was primarily adapted from previously validated instruments used in similar national and regional KAP surveys in Nigeria [13, 14]. Section A collected sociodemographic data; section B collected information on the knowledge of AMR; section C collected information on perceptions of AMR; and section D collected information on practices of AMS. Prior to the main study, the questionnaire was pretested for clarity, comprehension, and flow in a pilot involving 10% of the calculated sample size (43 health workers) from PHCs in Calabar metropolis not included in the main sampling frame. These pilot participants had similar demographics to the target population but were excluded from the final study sample to avoid bias.

Face validity was assessed by reviewing the questionnaire with three experts in public health. Content validity was ensured by aligning items with WHO guidelines on AMR knowledge and stewardship principles. Feedback from the pilot testing led to minor revisions, including simplification of ambiguous terms and rewording of double-barreled questions in the knowledge and perception sections. These modifications enhanced clarity and comprehension without altering the underlying constructs measured. Cronbach’s alpha for internal consistency was > 0.7 in key sections post-pilot. Specifically, the knowledge section had a Cronbach’s Alpha of 0.73, perceptions had 0.79, and practices had 0.75.

### Survey administration

Data were collected by independent data collectors who were public health graduates with prior experience in survey-based research. The data collectors were not employees of the participating health facilities and had no supervisory or professional authority over the respondents. All data collectors received standardised training on the study objectives, questionnaire administration, research ethics, informed consent, and techniques to minimise interviewer bias. In each healthcare facility, the eligible HWs were approached and informed about the study objectives. We minimised social desirability bias by training independent data collectors to use a neutral, standardised script and non-leading probes. Interviews were conducted in a private area without supervisors present. Participants were assured of anonymity and told clearly that responses would not affect employment or supervision. No names or personal identifiers were collected and questionnaires carried only study identification numbers. Sensitive items were read verbatim with neutral wording and consenting HWs could point to response options on a card.

A structured questionnaire was both interviewer-administered and self-administered (for respondents who had smartphones where questionnaire links could be sent) to each consenting healthcare practitioner, and entered directly into open data kit collect (ODK). In cases where consenting HWs and the researchers experienced challenges with internet access, the survey was administered to them using a paper-based questionnaire, which was later entered into the electronic app. Study participation was explained to the HWs to be anonymous, confidential and voluntary, and the HWs could withdraw at any time. The data collection process was completed within a three-week period.

### Method of data analysis

Data were entered into Microsoft Excel 2016 and checked for completeness. Data were then transferred to Statistical Products and Service Solutions (SPSS) Version 23 for analysis. Descriptive statistics (means, standard deviations, and frequency tables) and Chi-square were used. Chi square was used to test the hypotheses. Twenty-four knowledge questions were assessed with ‘yes’ or ‘no’ responses, where ‘yes’ indicated the correct answer and ‘no’ an incorrect answer. Each correct response scored 1, and incorrect responses scored 0, with a minimum score of 0 and a maximum of 24. Using a modified Bloom’s cut-off [18], knowledge was categorised as follows: ≥80% (≥ 19/24 points) as “good knowledge,” 50–79% (12–18 points) as “moderate knowledge,” and < 50% (< 12 points) as “poor knowledge.” The 80% cut-off assumes HWs have access to AMR training and information sources, reflecting an expectation of high proficiency in this critical area for healthcare professionals.

Perceptions were assessed using a 35-item questionnaire with a 5-point Likert scale: Strongly disagree (1), Disagree (2), Neutral (3), Agree (4), and Strongly agree (5). The minimum and maximum perception scores were 35 and 175, respectively. Items 14, 23, 25, 26, 33, and 35 were reverse-coded for consistent directionality, with higher scores indicating positive perceptions toward AMS . A conceptual cut-off was applied. A total score greater than 105 corresponded to an average item score above 3 (“neutral”), indicating overall agreement and positive perceptions toward antimicrobial use and resistance. Scores of 105 or below were therefore classified as poor perceptions. This cut-off was defined a priori, before data collection, to distinguish neutral or negative perceptions from clearly positive perceptions. Practices were evaluated using a 33-item questionnaire. Appropriate practices scored 1, while inappropriate or non-practiced actions scored 0. A Bloom’s cut-off of ≥ 70% (≥ 23/33 points) denoted “good practices,” and < 70% (< 23 points) denoted “poor practices.” The lower threshold accounted for systemic barriers like resource constraints and patient pressure. Chi-square tests of independence were used to assess associations between categorical variables (knowledge, perceptions, and practices vs. professional role and years of working experience), where Fisher’s exact test applied assumption of minimum expected cell counts were violated. The significance threshold was set at α = 0.05. *p* < 0.05.

## Results

Of 431 HWs approached, 427 responded, thus yielding a 99% response rate.

### Demographic characteristics of consenting HWs

Table 1 shows that Nurses constituted the largest professional group (37.0%), followed by Medical Doctors (26.5%) and Pharmacists (10.3%). Community health cadres (CHEW and JCHEW) accounted for 11.2% of respondents, while laboratory personnel represented 8.7%.). Consenting HWs with greater than 20 years of experience were the least represented in the study.

**Table 1:** Sociodemographic characteristics of respondents

Variables	Frequency (*n* = 427)	Per cent %
**Role**		
Nurse	158	37.0
Medical Doctor	113	26.5
Pharmacist	44	10.3
CHEW	39	9.1
Lab Scientist	25	5.9
Pharmacy Technician	14	3.3
Medical Microbiologist	12	2.8
JCHEW	9	2.1
Health Assistant	8	1.9
Health Supervisor	5	1.2
**Years of experience**		
Less than 1 year	30	7.0
1–4 years	100	23.4
5–9 years	120	28.1
10–14 years	90	21.1
15–20 years	60	14.1
Greater than 20 years	27	6.3

### HWs’ knowledge of antimicrobial use and AMR

**Fig. 2:**
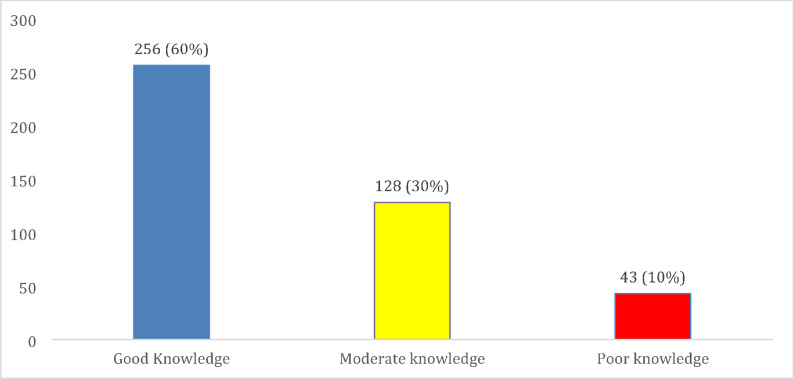
Knowledge level of HWs on antimicrobial use and AMR in Cross River State

Table 2 shows knowledge question responses. A significant majority (95.0%, *n* = 406) correctly identified that antibiotics are effective against bacteria, and 92.0% (*n* = 393) recognised that antibiotics are not effective against viruses. Additionally, 89.9% (*n* = 384) accurately defined AMR, and 92.0% (*n* = 393) acknowledged that bacteria can develop resistance to antimicrobials. Regarding AMR causes, 88.1% (*n* = 376) identified overuse of antibiotics, and 86.0% (*n* = 367) recognised poor infection prevention and control as contributors. Strategies to tackle AMR, such as developing institutional guidelines (95.0%, *n* = 406) and implementing infection prevention and control measures (88.1%, *n* = 376), were widely acknowledged. However, only 65.1% (*n* = 278) recognised the importance of investing in research into discovering new antimicrobial medicines. Figure 2 shows that 60.0% had good knowledge, 30.0% moderate, and 10.0% poor knowledge.

**Table 2:** HWs’ knowledge of antimicrobial use and AMR (*n* = 427)

Item	Yes *N* (%)	No *N* (%)
1. Antibiotics are effective against bacteria	406 (95.0)^#^	21 (5.0)
2. Antibiotics are effective against viruses	34 (8.0)	393 (92.0) ^#^
3. Antibiotics are effective against fungi	43 (10.1)	384 (89.9) ^#^
4. Antibiotics are effective against parasites	51 (11.9)	376 (88.1) ^#^
5. Definition of AMR t correctly answered	384 (89.9)	43 (10.1)
6. Bacteria can become resistant to antimicrobials	393 (92.0) ^#^	34 (8.0)
7. Viruses can become resistant to antimicrobials	85 (19.9) ^#^	342 (80.1)
8. Fungi can become resistant to antimicrobials	350 (82.0) ^#^	77 (18.0)
9. Parasites can become resistant to antimicrobials	338 (79.2) ^#^	89 (20.8)
10. Humans can become resistant to antimicrobials	34 (8.0)	393 (92.0) ^#^
11. Animals can become resistant to antimicrobials	384 (89.9) ^#^	43 (10.1)
12. Causes of AMR: Poor infection prevention and control	367 (86.0) ^#^	60 (14.0)
13. Causes of AMR: Inadequate hand hygiene	363 (85.0) ^#^	64 (15.0)
14. Causes of AMR: Use of antibiotics	351 (82.2) ^#^	76 (17.8)
15. Causes of AMR: Overuse of antibiotics	376 (88.1) ^#^	51 (11.9)
16. Tackling AMR: Surveillance	342 (80.1) ^#^	85 (19.9)
17. Tackling AMR: Public awareness	346 (81.0) ^#^	81 (19.0)
18. Tackling AMR: Healthcare professional training	350 (82.0) ^#^	77 (18.0)
19. Tackling AMR: Infection Prevention and Control	376 (88.1) ^#^	51 (11.9)
20. Tackling AMR: Antimicrobial Stewardship	363 (85.0) ^#^	64 (15.0)
21. Tackling AMR: Investment in new medicines	278 (65.1) ^#^	149 (34.9)
22. Tackling AMR: Development of institutional guidelines	406 (95.0) ^#^	21 (5.0)
23. Tackling AMR: Audit and feedback	307 (71.9) ^#^	120 (28.1)
24. Correctly ordered the WHO antibiotic categories (Access, Reserve, Watch) order of use	342 (80.1) ^#^	85 (19.9)

^#=^ Correct answers

### HWs’ perceptions on antimicrobial use and AMR

Table 3 shows perceptions of the HWs on AMR and antimicrobial use. A noTable 64.4% (*n* = 275) either agreed (47.3%, *n* = 202) or strongly agreed (17.1%, *n* = 73) that they are worried about AMR, and 75.4% (*n* = 322) agreed or strongly agreed that AMR is an important issue in their daily practice. Additionally, 54.1% (*n* = 231) believed their actions could protect antimicrobial effectiveness, and 79.2% (*n* = 338) felt that everyone can promote AMR awareness. Compliance with prescribing guidelines was seen as effective in preventing AMR by 63.2% (*n* = 270), though only 36.5% (*n* = 156) strongly agreed that guideline compliance prevents AMR. Hand hygiene was endorsed as a preventive measure, with 55.5% (*n* = 237) agreeing or strongly agreeing. However, confidence in challenging inappropriate prescribing was low, with 65.1% (*n* = 278) strongly disagreeing that they felt confident doing so. Furthermore, 60.2% (*n* = 257) strongly agreed that easy access to antibiotics without a prescription contributes to AMR, and 89.0% (*n* = 380) agreed or strongly agreed that AMR is a significant problem in their hospital, underscoring localised concerns about AMR prevalence and management. A total of 347 (81.4%) had good perceptions while 80 (18.6%) had poor perceptions on antimicrobial use and AMR among HWs.

**Table 3:** HWs’ perceptions on antimicrobial use and AMR

Items	SD *n* (%)	D *n* (%)	*N* *n* (%)	A *n* (%)	SA *n* (%)
1. I am worried about AMR	16 (3.7)	118 (27.6)	18 (4.2)	202 (47.3)	73 (17.1)
2. AMR is an important issue in my daily practice	39 (9.1)	1 (0.2)	65 (15.2)	264 (61.8)	58 (13.6)
3. My actions can protect antimicrobial effectiveness	141 (33.0)	18 (4.2)	37 (8.7)	134 (31.4)	97 (22.7)
4. Everyone can promote AMR awareness	99 (23.2)	14 (3.3)	86 (20.1)	201 (47.1)	27 (6.3)
5. Prescribing guidelines support preventing AMR	66 (15.5)	57 (13.3)	1 (0.2)	168 (39.3)	135 (31.6)
6. Prescribing guidelines are easy to implement	148 (34.7)	26 (6.1)	97 (22.7)	135 (31.6)	21 (4.9)
7. Guideline compliance prevents AMR	6 (1.4)	14 (3.3)	137 (32.1)	114 (26.7)	156 (36.5)
8. Hand hygiene prevents infection and AMR	84 (19.7)	34 (8.0)	72 (16.9)	43 (10.1)	194 (45.4)
9. I am motivated to advocate against AMR	9 (2.1)	59 (13.8)	13 (3.0)	314 (73.5)	32 (7.5)
10. Sharing AMR knowledge improves practice	25 (5.9)	18 (4.2)	58 (13.6)	130 (30.4)	196 (45.9)
11. Challenging inappropriate antimicrobial use is important	4 (0.9)	117 (27.4)	29 (6.8)	186 (43.6)	91 (21.3)
12. I am confident in challenging inappropriate prescribing	278 (65.1)	1 (0.2)	28 (6.6)	45 (10.5)	75 (17.6)
13. Advocating for infection prevention is key to AMS	52 (12.2)	114 (26.7)	120 (28.1)	93 (21.8)	48 (11.2)
14. Patients take antibiotics inappropriately regardless of my actions	110 (25.8)	15 (3.5)	30 (7.0)	189 (44.3)	83 (19.4)
15. Advising patients/public about AMR is important	30 (7.0)	112 (26.2)	101 (23.7)	111 (26.0)	73 (17.1)
16. Quantifying antimicrobial use identifies AMS gaps	25 (5.9)	58 (13.6)	80 (18.7)	123 (28.8)	141 (33.0)
17. I consider AMR when treating a patient	93 (21.8)	53 (12.4)	11 (2.6)	61 (14.3)	209 (48.9)
18. AMR is a significant problem worldwide	0 (0.0)	28 (6.6)	242 (56.7)	109 (25.5)	48 (11.2)
19. AMR is a significant problem in my country	82 (19.2)	149 (34.9)	1 (0.2)	148 (34.7)	47 (11.0)
20. AMR is a significant problem in my hospital	92 (21.5)	50 (11.7)	26 (6.1)	258 (60.4)	1 (0.2)
21. Easy access to antibiotics without a prescription contributes to AMR	6 (1.4)	52 (12.2)	30 (7.0)	82 (19.2)	257 (60.2)
22. My institution performs adequate surveillance for resistant organisms	0 (0.0)	177 (41.5)	77 (18.0)	136 (31.9)	37 (8.7)
23. Lack of diagnostic tests leads to antimicrobial overuse	9 (2.1)	22 (5.2)	127 (29.7)	118 (27.6)	151 (35.4)
24. My institution provides adequate AMR education	105 (24.6)	189 (44.3)	22 (5.2)	79 (18.5)	32 (7.5)
25. I suspect antimicrobials in my institution are of poor quality	77 (18.0)	61 (14.3)	69 (16.2)	160 (37.5)	60 (14.1)
26. Sporadic antimicrobial supply leads to therapy interruptions	17 (4.0)	110 (25.8)	30 (7.0)	217 (50.8)	53 (12.4)
27. Cost considerations affect my antimicrobial choice	11 (2.6)	108 (25.3)	25 (5.9)	222 (52.0)	61 (14.3)
28. AMS improves the quality of patient care	104 (24.4)	57 (13.3)	43 (10.1)	51 (11.9)	172 (40.3)
29. AMS reduces antibiotic use overall	100 (23.4)	41 (9.6)	212 (49.6)	45 (10.5)	29 (6.8)
30. AMS reduces hospital stay and costs	35 (8.2)	40 (9.4)	58 (13.6)	174 (40.7)	120 (28.1)
31. My institution can implement an effective AMS program	53 (12.4)	43 (10.1)	58 (13.6)	98 (22.9)	175 (41.0)
32. My institution has the capacity for effective AMS	38 (8.9)	40 (9.4)	40 (9.4)	124 (29.0)	185 (43.3)
33. AMS can be an obstacle to good patient care	58 (13.6)	15 (3.5)	97 (22.7)	139 (32.6)	118 (27.6)
34. Infectious disease experts are available for guidance	22 (5.2)	3 (0.7)	157 (36.8)	124 (29.0)	121 (28.3)
35. Only prescribing physicians need to understand AMS	109 (25.5)	51 (11.9)	47 (11.0)	181 (42.4)	39 (9.1)

SD= Strongly Disagree; D= Disagree; N=Neutral; A= Agree; SA= Strongly agree

### HWs’ practices relating to antimicrobial use and AMR

Table 4 presents the practices of HWs related to AMR and antimicrobial use. A substantial proportion of the HWs 87.1% (*n* = 372) reported advising colleagues on the appropriate use of antimicrobials, and 82.0% (*n* = 350) felt confident advising patients on antibiotic use. Additionally, 90.9% (*n* = 388) were aware of safe antimicrobial disposal protocols, and 79.9% (*n* = 341) said they referred to standard treatment guidelines (STG) before prescribing. However, concerning practices were also prevalent, with 54.3% (*n* = 232) admitting to supplying antibiotics not in accordance with guidelines, and 65.8% (*n* = 281) determining formulation and dose based on stock availability rather than guidelines. Furthermore, 46.8% (*n* = 200) prescribed antibiotics due to patient pressure, and 46.4% (*n* = 198) provided broader-spectrum antibiotics due to doubts about suitability. On a positive note, 72.8% (*n* = 311) of those queried prescriptions lacking evidence of infection, and 76.6% (*n* = 327) were certain of protocols to follow when concerned. A total of 277 (65%) of HWs had good practices relating to antimicrobial use and AMR while 150 (35%) did not.

**Table 4:** HWs’ practices relating to antimicrobial use and AMR

Practices	Frequency (*n*)	% of respondents
1. Supplied an antibiotic not first recommended due to limited stock	145	34.0
2. Provided less than the recommended dose of an antibiotic	145	34.0
3. Queried a prescription due to insufficient evidence of infection	311	72.8
4. Had doubts on the efficacy of the antimicrobial batch	215	50.4
5. Reported/sent for testing an antimicrobial batch I doubted	304	71.2
6. Supplied an antibiotic not in line with guidelines	232	54.3
7. Advised a colleague on the most appropriate antimicrobial	372	87.1
8. Safely disposed of antimicrobials at work	248	58.1
9. Contributed to AMS strategies at my workplace	296	69.3
10. Involved in collecting data on AMR	372	87.1
11. Followed up with a patient supplied with an antimicrobial	294	68.9
12. Referred to the Standard Treatment Guideline (STG) for a prescribed antimicrobial	260	60.9
13. Formulation and dose provided were determined by stock rather than guidelines	281	65.8
14. Worried about the quality of antibiotic formulations and their impact on care	380	89.0
15. Sure of protocol to check with prescriber when concerned about a prescription	327	76.6
16. Prescriber expects me to query concerning prescriptions	246	57.6
17. Check antibiotic choice with a peer or superior when uncertain	317	74.2
18. Felt pressure to supply antibiotics when not clinically required	161	37.7
19. Confident in independently supplying for infection treatment without a prescription	171	40.0
20. Confident in advising patients on how to use/take antibiotics	350	82.0
21. Aware of how antimicrobials can be safely disposed of at work	388	90.9
22. My role includes contributing to the hospital’s AMR goals	347	81.3
23. My role includes collecting data to support tackling AMR	321	75.2
24. My role includes giving feedback to colleagues about antimicrobial use	333	78.0
25. Provided an antibiotic due to fear of patient deterioration	160	37.5
26. Provided multiple antimicrobials to the same patient	259	60.7
27. Stopped an antibiotic supply earlier than prescribed	137	32.1
28. Checked my choice with a senior colleague	330	77.3
29. Prescribed an antibiotic due to patient pressure to maintain the relationship	200	46.8
30. Prescribed an antibiotic due to uncertainty about the infection diagnosis	144	33.7
31. Provided a broad/wider spectrum antibiotic due to doubts about suitability	198	46.4
32. Received feedback on my antimicrobial prescribing	246	57.6
33. Referred to the Standard Treatment Guideline before prescribing an antimicrobial	341	79.9

### Test of hypotheses

There is no statistically significant association between HWs’ socio-demographic characteristics and their knowledge of antimicrobial use and AMR.

There were statistically significant differences in knowledge of antimicrobial use and AMR across professional cadres (Table 5).

**Table 5:** Chi-square for association between knowledge of AMR and demographic characteristics (*n* = 427)

	Poor *n* (%)	Knowledge levels			
		Moderate *n* (%)	Good *n* (%)	χ²	*p*-Value
**Role**				41.72	0.001*
CHEW	8 (20.5)	17 (43.6)	14 (35.9)		
Health Assistant	2 (25.0)	3 (37.5)	3 (37.5)		
Health Supervisor	0 (0.0)	2 (40.0)	3 (60.0)		
JCHEW	3 (33.3)	3 (33.3)	3 (33.3)		
Lab Scientist	3 (12.0)	7 (28.0)	15 (60.0)		
Medical Microbiologist	2 (16.7)	3 (25.0)	7 (58.3)		
Medical Doctor	9 (8.0)	31 (27.4)	73 (64.6)		
Nurse	11 (7.0)	43 (27.2)	104 (65.8)		
Pharmacist	5 (11.4)	14 (31.8)	25 (56.8)		
Pharmacy Technician	2 (14.3)	5 (35.7)	7 (50.0)		
**Years of experience**				26.78	0.013*
Less than 1 year	5 (16.7)	12 (40.0)	13 (43.3)		
1–4 years	13 (13.0)	34 (34.0)	53 (53.0)		
5–9 years	12 (10.0)	36 (30.0)	72 (60.0)		
10–14 years	7 (7.8)	27 (30.0)	56 (62.2)		
15–20 years	4 (6.7)	17 (28.3)	39 (65.0)		
Greater than 20 years	2 (7.4)	7 (25.9)	18 (66.7)		

χ²= Chi-square statistic; P-value= Probability value; *=Statistical significance (*P* < 0.05)

Table 5 shows a significant association between role and knowledge level (χ²=41.72, df = 18, *p* = 0.001). Nurses (65.8%) and Medical Doctors (64.6%) had higher proportions of good knowledge. Cramer’s V (0.221, *p* = 0.001) indicates a small-to-moderate effect of respondents’ roles on their knowledge of AMR and antimicrobial use. Years of experience were significant (χ²=26.78, df = 10, *p* = 0.013), with > 20 years showing higher knowledge. Cramer’s V (0.177, *p* = 0.013) suggests a small but statistically significant effect of years of experience on knowledge of AMR and antimicrobial use.

.

In contrast, no statistically significant associations were observed between professional cadre or years of experience and perceptions toward antimicrobial use and AMR (Table 6). The proportion of respondents with good perceptions was similar across cadres (*p* = 0.171) and experience categories (*p* = 0.418).

**Table 6:** Chi-Square for association between HWs’ perceptions on AMR and sociodemographic characteristics (*n* = 427)

	Perceptions			
	Poor *n* (%)	Good *n* (%)	χ²	*p*-Value
**Role**			12.84	0.171
CHEW	4 (10.3)	35 (89.7)		
Health Assistant	2 (25.0)	6 (75.0)		
Health Supervisor	2 (40.0)	3 (60.0)		
JCHEW	3 (33.3)	6 (66.7)		
Lab Scientist	4 (16.0)	21 (84.0)		
Medical Microbiologist	2 (16.7)	10 (83.3)		
Medical Doctor	20 (17.7)	93 (82.3)		
Nurse	19 (12.0)	139 (88.0)		
Pharmacist	8 (18.2)	36 (81.8)		
Pharmacy Technician	3 (21.4)	11 (78.6)		
**Years of experience**			4.98	0.418
Less than 1 year	3 (10.0)	27 (90.0)		
1–4 years	21 (21.0)	79 (79.0)		
5–9 years	28 (23.3)	92 (76.7)		
10–14 years	20 (22.2)	70 (77.8)		
15–20 years	9 (15.0)	51 (85.0)		
Greater than 20 years	4 (14.8)	23 (85.2)		

χ²= Chi-square statistic; P-value= Probability value

Practices related to antimicrobial use and AMR differed significantly by professional cadre and years of experience (Table 7). Nurses demonstrated a higher proportion of good practices compared with other cadres (χ² = 28.41, df = 9, *p* = 0.001). Additionally, HWs with more than 20 years of experience were more likely to report good AMS practices compared with those with fewer years of experience (χ² = 14.67, df = 5, *p* = 0.012).

**Table 7:** Chi-square for association between practices of HWs relating to AMR and antimicrobial use and sociodemographic characteristics (*n* = 427)

	Practices			
	Poor *n* (%)	Good *n* (%)	χ²	*p*-Value
**Role**				
CHEW	24 (61.5)	15 (38.5)		
Health Assistant	5 (62.5)	3 (37.5)		
Health Supervisor	3 (60.0)	2 (40.0)		
JCHEW	5 (55.6)	4 (44.4)		
Lab Scientist	10 (40.0)	15 (60.0)		
Medical Microbiologist	5 (41.7)	7 (58.3)		
Medical Doctor	32 (28.3)	81 (71.7)		
Nurse	41 (25.9)	117 (74.1)	28.41	0.001*
Pharmacist	16 (36.4)	28 (63.6)		
Pharmacy Technician	6 (42.9)	8 (57.1)		
**Years of experience**				
Less than 1 year	18 (60.0)	12 (40.0)		
1–4 years	46 (46.0)	54 (54.0)		
5–9 years	47 (39.2)	73 (60.8)		
10–14 years	28 (31.1)	62 (68.9)		
15–20 years	18 (30.0)	42 (70.0)		
Greater than 20 years	7 (25.9)	20 (74.1)	14.67	0.012*

χ²= Chi-square statistic; P-value= Probability value; *=Statistical significance (*P* < 0.05)

## Discussion

This study showed that knowledge and perceptions relating to antimicrobial use and resistance among this cohort of HWs in CRS were generally good as most consenting HWs.

scored well on knowledge (60% “good”) and held positive perceptions (81.4% “good”). However, practice was constrained by stock availability and patient pressure (between 47 and 66%). It highlights opportunities for HWs to mitigate AMR in an LMIC setting.

### Knowledge of antimicrobial use and AMR

Most of the HWs had good knowledge of AMR and antimicrobial use (60% and above). This level is different from the 70% found among healthcare professionals in Zambia [19]. Similarly, AMR knowledge was found to be significantly high (66.1% − 88%) among HWs in the Benin Republic [20]. A study in Niger State of Nigeria [21] had similar the findings of this present study, with 62.3% of healthcare professionals were found to have good knowledge of AMR and antimicrobial use. In neighboring Akwa Ibom State, however, there was a notable deficit in knowledge of AMR and WHO Access, Watch, Reserve (AwaRe) classification among healthcare providers, reflecting regional variation guided by access to training [22]. However, common misconceptions persist, as shown in the current data where participants incorrectly believed that antibiotics are effective against viral infections, and some considered antibiotics appropriate for parasitic infections. These are in line with broader global trends [23]. This is typically driven by inadequate education on microbial dynamics [21, 22]. We found that 54.3% of the prescribers supplied antibiotics not in line with guidelines which suggests that practice lags behind what people know and believe. Good knowledge of AMR among HWs is a result of increased awareness campaigns, targeted educational interventions, National Action Plans, the widely adopted One Health Approach, and international interventions [14]. The knowledge gaps reported in this study and others [22–24] highlight the need for educational interventions to increase knowledge and attitudes. These educational initiatives should address the knowledge gaps identified such as highlighting the crucial role of hand hygiene, how resistance to antimicrobials develop and the critical need for more research into the discovery of new antimicrobial agents. After discussing the survey data by professional cadre, potential options include in-venue or online training, educational outreach, opinion leaders, peer groups, and prescription review (clinical audit) among health care professionals, with targeted outreaches to different cadres [25].

The robust association between professional status (e.g., physicians and nurses having higher knowledge) and years of experience (> 20 years translating to better scores) shows that specialist training and experiential learning enhance understanding. This is consistent with findings from studies in which healthcare professionals’ knowledge was poor but improved with increasing practice years, and with emphasis being placed on professional continuous development [26, 27]. In turn, CHEWs can lag behind due to limited formal education, a not unusual scenario in primary care settings in Africa. The robust association between professional cadre (e.g., physicians and nurses having higher knowledge) and years of experience (> 20 years translating to better scores) shows that specialist training and experiential learning enhance understanding of AMR concepts [28].

### Perceptions on antimicrobial use and AMR

Perceptions of HWs on AMR and antimicrobial use were significantly positive, with some agreement over the efficacy of preventive measures such as hand hygiene and guideline adherence. However, only 55% agreed or strongly agreed that hand hygiene is useful for preventing AMR. This highlights another gap in knowledge that points to the need for more awareness-raising and training on this issue. Such positivity is corroborated by studies [14, 29, 30] and is evidence of growing awareness, perhaps spurred locally by national efforts such as under Nigeria’s AMR Action Plan. Perceptions in the African setting tend to follow with institutional support; a regional review [31] revealed gaps in KAP due to system-level reasons like an absence of resources, which could explain the lack of demographic associations in the current study.

In contrast to practices and knowledge, perceptions were not significantly associated with experience or role, indicating that they may be more affected by the outside world, such as media and policy exposure rather than profession.

### Practices on antimicrobial use and AMR

While 65% of the reported practices were good, system vulnerabilities are expressed via alarming practices like unethical prescribing driven by external non-clinical factors, such as financial incentives, and patient pressure. These behaviours perpetuate AMR as exemplified by the prevalence of high broad-spectrum antibiotic prescribing due to diagnostic challenges [31–33], an issue prevalent in Nigerian health centers with limited training (e.g. CHEWs) and limited laboratories. Comparative studies in Nigeria and across Africa demonstrate similar problems, with only 45–51% having good prescribing practices, due often to health financing reasons and patient pressures [34]. Association with experience and role (nurses showing enhanced practice) indicates the significance of seniority in coping with these pressures. Interestingly, the nurses appeared to have enhanced practice with respect to AMS/AMR as opposed to doctors. It might be because the nurses are more inclined to follow antimicrobial policy guidelines as opposed to doctors who may vary their prescribing practices based on several other factors. Further research is required to fully understand this trend.

The practices-knowledge/perceptions differences in this study highlight the “know-do” gap [35, 36], whereby positive perceptions are not being converted into meaningful practices due to impediments like workload and stockouts. Stock-based prescribing such as giving a broad-spectrum antibiotic when a narrow-spectrum or required antibiotic is out of stock-further exacerbates the issue.

The widespread access to antibiotics through informal markets and direct purchase from pharmacies is a significant issue in Nigeria which can influence the perceptions and attitudes of HWs in several ways. When patients routinely obtain antibiotics without prescriptions, HWs may perceive a reduced level of control over antimicrobial use, leading to frustration and a sense of diminished professional authority in guiding appropriate treatment. This environment can normalise inappropriate antibiotic use, potentially lowering prescribers’ risk perception of misuse and reinforcing the belief that strict adherence to guidelines is impractical in real-world settings. In addition, HWs may feel pressured to prescribe antibiotics to align with patients’ prior self-medication practices or expectations, in order to maintain patient satisfaction and trust. Over time, such pressures can foster more permissive attitudes toward non-evidence-based prescribing and weaken commitment to AMS, thereby indirectly sustaining inappropriate antibiotic use and contributing to the escalation of AMR.

While it is vital to improve the training of HWs with the hope that this will improve their clinical practice, this needs to be complemented by other effective strategies. These include: implementation of clear treatment guidelines and decision-support tools, establishment of AMS teams or focal persons, strengthening infection diagnostic capacity as well as audit, feedback, and supervision with respect to antimicrobials.

### Implications for policy and practice in cross river state and beyond

In general, the study suggests that multi-dimensional interventions are required to reconcile practice with knowledge and perceptions. This makes a case for integrating AMR modules within pre-service training curriculum, with enhanced supply chains, and for interprofessional team working to counteract external pressures. Specifically, both pre-service and in-service training modules are required, including realistic case study exercises for skills development. Face-to-face training is likely preferable for in-service, but online training offers greater reach and scale-up at lower cost compared to venue-based sessions. Other options include peer groups facilitated by senior respected HWs/supervisors, which can incorporate prescription review (also called clinical audit). More targeted training needs to be channeled to junior-level staff, such as CHEWs and Junior CHEWs, to improve their knowledge and corresponding practices regarding AMR.

### Limitations

This study has some limitations. Self-reported bias may overestimate positive responses, and a cross-sectional design limits causality. The focus on public facilities may limit the generalisability of findings to the private sector. Subsequent studies need to employ mixed methods approaches, such as prescribing audits and longitudinal surveys, to track AMS impacts. The dichotomisation of practice scores into “good” and “poor” using a predefined cut-off may have obscured small but potentially meaningful differences in practice levels around the threshold. We chose this approach to account for systemic barriers and to clearly spot those with clearly poor practices, but we recognise it gives less detail than the three-level system used for knowledge. Additionally, the demographic data were limited to professional cadre and years of experience; information on gender, educational level, facility type, and prior AMS training was not collected, limiting fuller characterization of the sample. Qualitative studies are needed to better understand the reasons for the responses given in our survey, and to gather suggestions for how to improve knowledge, perceptions and practices of HWs with respect to antimicrobial use in this context. Also, interventions to improve AMS should be pilot-tested e.g. with pre/post training tests, and refined, prior to scale-up. Supervisors need to be involved in trainings, and follow-up visits to guide/ support change in AMS practice.

## Conclusion

Our study underscores the potential role of HWs in combating AMR in CRS, Nigeria, and highlights the intricate interplay of strengths and weaknesses in their knowledge, perceptions, and practices. The generally good knowledge and positive perceptions among HWs indicate a good platform for AMS, fueled by awareness of AMR’s global and local consequences. However, the gap in practices highlights systemic barriers undermining effective AMS. The results align with overall trends in LMICs, where resource and time constraints, and patient expectation pressures often undermine the translation of knowledge into practice.

The significant associations between professional cadres (doctors and nurses) and years of experience, along with better knowledge and practices, suggest that targeted interventions can utilise experienced HWs to mentor others in lower cadres.

## Data Availability

The datasets used and/or analysed during the current study will be available from the corresponding author on reasonable request.

## References

[1] Salam MA, Al-Amin MY, Salam MT, Pawar JS, Akhter N, Rabaan AA et al. Antimicrobial resistance: A growing serious threat for global public health. Healthc (Basel). 2023;11(13): 1946. 10.3390/healthcare11131946 PMC10340576 37444780

[2] Oliveira M, Antunes W, Mota S, Madureira-Carvalho Á, Dinis-Oliveira RJ. Dias Da Silva D. An overview of the recent advances in antimicrobial resistance. Microorganisms. 2024;12(9).: 1920 10.3390/microorganisms12091920 PMC11434382 39338594

[3] World Health Organization. Antimicrobial Resistance 2023 [Available from: https://www.who.int/news-room/fact-sheets/detail/antimicrobial-resistance

[4] Ehsan H. Antibiotic resistance in developing countries: emerging threats and policy responses. Public Health Challenges. 2025;4(1):e70034.

[5] Saleem Z, Mekonnen BA, Orubu ES, Islam MA, Nguyen TTP, Ubaka CM et al. Current access, availability and use of antibiotics in primary care among key low- and middle-income countries and the policy implications. Expert Rev Anti-infective Therapy 2025.1–42. 10.1080/14787210.2025.2477198 40110804

[6] Sharma S, Chauhan A, Ranjan A, Mathkor DM, Haque S, Ramniwas S et al. Emerging challenges in antimicrobial resistance: implications for pathogenic microorganisms, novel antibiotics, and their impact on sustainability. Front Microbiol. 2024; 15:1403168. 10.3389/fmicb.2024.1403168 PMC11089201 38741745

[7] World Health Organization. Antimicrobial resistance, key facts. Geneva, Switzerland: World Health Organization; 2020.

[8] Nigeria Centre for Disease Control and Prevention. Antimicrobial Resistance 2023 [Available from: https://ncdc.gov.ng/diseases/factsheet/70#:~:text=AMR%20occurs%20naturally%20over%20time,of%20antibiotic%20prescriptions%20at%2048%25

[9] Achi CR, Ayobami O, Mark G, Egwuenu A, Ogbolu D, Kabir J. Operationalising one health in nigeria: reflections from a High-Level expert panel discussion commemorating the 2020 world antibiotics awareness week. Front Public Health. 2021; 9:673504 10.3389/fpubh.2021.673504 PMC8203202 34136458

[10] Martak D, Henriot CP, Hocquet D. Environment, animals, and food as reservoirs of antibiotic-resistant bacteria for humans: one health or more? Infect Dis now. 2024;54(4):104895. 38548016 10.1016/j.idnow.2024.104895

[11] Nigeria Centre for Disease Control and Prevention. One Health ANTIMICROBIAL RESISTANCE National Action Plan 2.0 2024 2028 2024 [Available from: https://ncdc.gov.ng/themes/common/docs/protocols/353_1729270476.pdf

[12] Gashegu M, Gahamanyi N, Ndayambaje FX, Munyemana JB, Ndahindwa V, Lukwago F et al. Exploring prescription practices: insights from an antimicrobial stewardship program at a tertiary healthcare Facility, Rwanda. Antibiot (Basel). 2024;13(6): 548. 10.3390/antibiotics13060548 PMC11200619 38927214

[13] Ogoina D, Iliyasu G, Kwaghe V, Otu A, Akase IE, Adekanmbi O, et al. Predictors of antibiotic prescriptions: a knowledge, attitude and practice survey among physicians in tertiary hospitals in Nigeria. Antimicrob Resist Infect Control. 2021;10(1):73. 33931108 10.1186/s13756-021-00940-9 PMC8086089

[14] Chukwu EE, Oladele DA, Enwuru CA, Gogwan PL, Abuh D, Audu RA, et al. Antimicrobial resistance awareness and antibiotic prescribing behavior among healthcare workers in nigeria: a National survey. BMC Infect Dis. 2021;21(1):22. 33413172 10.1186/s12879-020-05689-x PMC7792030

[15] Chukwu EE, Oladele DA, Awoderu OB, Afocha EE, Lawal RG, Abdus-salam I, et al. A National survey of public awareness of antimicrobial resistance in Nigeria. Antimicrob Resist Infect Control. 2020;9(1):72. 32434552 10.1186/s13756-020-00739-0 PMC7238560

[16] Enechukwu OH, Saka MJ. Perception of antibiotic misuse and awareness of antibiotic resistance among adults in delta state Nigeria. Discover Public Health. 2024;21(1):124.

[17] Chukwu EE, Oladele DA, Enwuru CA, Gogwan PL, Abuh D, Audu RA, et al. Antimicrobial resistance awareness and antibiotic prescribing behavior among healthcare workers in nigeria: a National survey. BMC Infect Dis. 2021;21(1):22. 33413172 10.1186/s12879-020-05689-x PMC7792030

[18] Ashebir W, Yimer B, Alle A, Teshome M, Teka Y, Wolde A. Knowledge, attitude, practice, and factors associated with prevention practice towards COVID-19 among healthcare providers in Amhara region, Northern ethiopia: A multicenter cross-sectional study. PLOS Global Public Health. 2022;2(4):e0000171. 36962177 10.1371/journal.pgph.0000171 PMC10021359

[19] Tembo N, Mudenda S, Banda M, Chileshe M, Matafwali S. Knowledge, attitudes and practices on antimicrobial resistance among pharmacy personnel and nurses at a tertiary hospital in Ndola, zambia: implications for antimicrobial stewardship programmes. JAC Antimicrob Resist. 2022;4(5):dlac107. 36226225 10.1093/jacamr/dlac107 PMC9549736

[20] Allabi AC, Agbo AG, Boya B, Mudenda S. Antimicrobial stewardship: knowledge and attitudes of pharmacy staff on antibiotic dispensing patterns, use and resistance in Benin. Pharmacol Pharm. 2023;14(6):189–214.

[21] Abubakar B, Sárváry A. Knowledge, attitude, and practice on antibiotics use among healthcare workers: A cross-sectional study in Niger state, Nigeria. J Infect Prev. 2023;24(5):206–15. 37736128 10.1177/17571774231165407 PMC10510657

[22] Akpan MR, Jackson IL, Eshiet UI, Mfon SA, Abasiattai EA. Knowledge of antimicrobial stewardship and the Access, watch and reserve (AWaRe) classification of antibiotics among frontline healthcare professionals in Akwa Ibom State, nigeria: a cross-sectional study. BMC Health Serv Res. 2024;24(1):1014. 39223650 10.1186/s12913-024-11428-8 PMC11370112

[23] Chalkidou A, Lambert M, Cordoba G, Taxis K, Hansen MP, Bjerrum L. Misconceptions and knowledge gaps on antibiotic use and resistance in four healthcare settings and five European Countries—A modified Delphi study. Antibiotics. 2023;12(9):1435. 37760731 10.3390/antibiotics12091435 PMC10525245

[24] Balliram R, Sibanda W, Essack SY. The knowledge, attitudes and practices of doctors, pharmacists and nurses on antimicrobials, antimicrobial resistance and antimicrobial stewardship in South Africa. S Afr J Infect Dis. 2021;36(1):262. 34485504 10.4102/sajid.v36i1.262 PMC8378097

[25] Davey P, Scott CL, Brown E, Charani E, Michie S, Ramsay CR, et al. Interventions to improve antibiotic prescribing practices for hospital inpatients (updated protocol). Cochrane Database Syst Rev. 2017;2017(2):CD011236. 10.1002/14651858.CD003543.pub4 PMC6464541 28178770

[26] Fuller W, Kapona O, Aboderin AO, Adeyemo AT, Olatunbosun OI, Gahimbare L, et al. Education and awareness on antimicrobial resistance in the WHO African region: a systematic review. Antibiotics. 2023;12(11):1613. 37998815 10.3390/antibiotics12111613 PMC10669252

[27] Kaniu MW, Gitaka WR, Jain R, Munyare AN, Adam RD, Monroe-Wise A. Knowledge, attitudes, and practices regarding antimicrobial resistance and antimicrobial stewardship among healthcare workers in outpatient medical centers in kenya: a qualitative study. Antimicrob Stewardship Healthc Epidemiol. 2025;5(1):e113. 10.1017/ash.2025.41 PMC12089738 40395948

[28] Jahromi AS, Namavari N, Jokar M, Sharifi N, Soleimanpour S, Naserzadeh N, et al. Global knowledge, attitudes, and practices towards antimicrobial resistance among healthcare workers: a systematic review and meta-analysis. Antimicrob Resist Infect Control. 2025;14(1):47. 40361230 10.1186/s13756-025-01562-1 PMC12076913

[29] Llor C, Bjerrum L. Antimicrobial resistance: risk associated with antibiotic overuse and initiatives to reduce the problem. Ther Adv Drug Saf. 2014;5(6):229–41. 25436105 10.1177/2042098614554919 PMC4232501

[30] Mesafint E, Wondwosen Y, Dagnaw GG, Gessese AT, Molla AB, Dessalegn B, et al. Study on knowledge, attitudes and behavioral practices of antimicrobial usage and resistance in animals and humans in Bahir Dar City, Northwest Ethiopia. BMC Public Health. 2024;24(1):2632. 39334050 10.1186/s12889-024-20110-x PMC11438306

[31] Ndihokubwayo JB, Yahaya AA, Desta AT, Ki-Zerbo G, Odei EA, Keita B, et al. Antimicrobial resistance in the African region: Issues, challenges and actions proposed. Afr Health Monit. 2013;16:27–30.

[32] Skender K, Machowska A, Khare S, Singh V, Lundborg CS, Sharma M. Antibiotic prescribing practices, perceived constraints, and views on antimicrobial resistance among general and orthopedic surgeons in central India. Sci Rep. 2025;15(1):25099. 40646238 10.1038/s41598-025-11173-w PMC12254354

[33] Otaigbe II, Elikwu CJ. Drivers of inappropriate antibiotic use in low- and middle-income countries. JAC Antimicrob Resist. 2023;5(3):dlad062. 37265987 10.1093/jacamr/dlad062 PMC10230568

[34] Saleem Z, Mekonnen BA, Orubu ES, Islam MA, Nguyen TTP, Ubaka CM et al. Current access, availability and use of antibiotics in primary care among key low- and middle-income countries and the policy implications. Expert Rev Anti-infective Therapy. 2025:1–42. 10.1080/14787210.2025.2477198 40110804

[35] Leonard KL, Masatu MC. Professionalism and the know-do gap: exploring intrinsic motivation among health workers in Tanzania. Health Econ. 2010;19(12):1461–77. 19960481 10.1002/hec.1564

[36] Lagarde M, Blaauw D. Levels and determinants of overprescribing of antibiotics in the public and private primary care sectors in South Africa. BMJ Global Health. 2023;8(7). 10.1136/bmjgh-2023-012374 PMC10391785 37524502

